# Weakly unconditionally Cauchy series and Fibonacci sequence spaces

**DOI:** 10.1186/s13660-017-1407-y

**Published:** 2017-06-08

**Authors:** Ramazan Kama, Bilal Altay

**Affiliations:** 1 Department of Mathematics, Siirt University, Siirt, Turkey; 2 Faculty of Education, Inonu University, Malatya, Turkey

**Keywords:** 46B15, 40A05, 46B45, Fibonacci sequence spaces, weakly unconditionally Cauchy series, completeness, barreledness

## Abstract

We study new sequence spaces associated to sequences in normed spaces and the band matrix *F̂* defined by the Fibonacci sequence. We give some characterizations of continuous linear operators and weakly unconditionally Cauchy series by means of completeness of the new sequence spaces. Also, we characterize the barreledness of a normed space via weakly^∗^ unconditionally Cauchy series in $X^{*}$.

## Introduction

By *w*, we denote the space of all real sequences $x=(x_{k})$. Any vector subspace of *w* is called a *sequence space*. We have $\ell_{\infty }$, *c* and $c_{0}$ for the spaces of all bounded, convergent and null sequences $x=(x_{k})$, respectively, normed by $\Vert x \Vert _{\infty }=\sup_{k}\vert x_{k} \vert $, where $k\in \mathbb{N}$, the set of positive integers.

A sequence space *λ* with a linear topology is called a *K-space* provided each of the maps $p_{i}:\lambda \to \mathbb{R}$ defined by $p_{i}(x)=x_{i}$ is continuous for all $i\in \mathbb{N}$. A K-space *λ* is called an *FK-space* provided *λ* is a complete linear metric space. We say that an FK space $\lambda \supset c_{00}$ has AD if $c_{00}$ is dense in *λ*, where $c_{00}=\operatorname{\operatorname{span}}\{e^{n}:n\in \mathbb{N}\}$, the set of all finitely non-zero sequences.

Let $A=(a_{nk})$ be an infinite matrix of real numbers $a_{nk}$, where $n,~k \in \mathbb{N}$. Then we write $Ax= ((Ax)_{n} )$, the *A*-transform of $x\in w$, if $(Ax)_{n}=\sum_{k} a_{nk}x_{k}$ converges for each $n\in \mathbb{N}$. For a sequence space *λ*, the matrix domain $\lambda_{A}$ of an infinite matrix *A* is defined by

$$\begin{aligned} \lambda_{A}= \bigl\{ x=(x_{k})\in w:Ax\in \lambda \bigr\} , \end{aligned}$$

which is a sequence space.

A series $\sum_{k}x_{k}$ in a real Banach space *X* is called weakly unconditionally Cauchy series (wuCs) if $\sum_{k}\vert f(x_{k}) \vert <\infty $ for every $f\in X^{*}$ (the dual space of *X*), and is called unconditionally convergent (ucs) if $\sum_{k}x_{\pi (k)}$ is convergent for every permutation *π* of $\mathbb{N}$. By $\operatorname{ucs}(X)$, $\ell_{1}(X)$, $\operatorname{cs}(X)$, $\operatorname{wcs}(X)$ and $\operatorname{wuCs}(X)$, we denote the *X*-valued sequence spaces of unconditionally convergent, absolutely convergent, convergent, weakly convergent and weakly unconditionally Cauchy series, respectively.

It is well known that (see [1] and [2]) that $x=(x_{k})\in \operatorname{ucs}(X)$ if and only if $(a_{k}x_{k})\in \operatorname{cs}(X)$ for every $a=(a_{k})\in l_{\infty }$, and $x=(x_{k})\in \operatorname{wuCs}(X)$ if and only if $(a_{k}x_{k})\in \operatorname{cs}(X)$ for every $a=(a_{k})\in c_{0}$. It is also well known (see [3] and [4]) that *X* has a copy of $c_{0}$ if and only if $\operatorname{wuCs}(X)\setminus \operatorname{ucs}(X)\neq \emptyset $, and if *X* is a normed space then $x=(x_{k})\in \operatorname{wuCs}(X)$ if and only if the set

1.1 $$\begin{aligned} E= \Biggl\{ \sum_{k=1}^{n}a_{k}x_{k}: \, \vert a_{k} \vert \leq 1,\,k=1,2,\ldots ,n; \, n\in \mathbb{N} \Biggr\} \end{aligned}$$

is bounded. Another characterization of weakly unconditionally Cauchy series that appears in [1] states that a sequence $x=(x_{k})$ is in $\operatorname{wuCs}(X)$ if and only if there is a bounded operator $T:c_{0} \rightarrow X$ defined by $T(a)=\sum_{k} a_{k}x_{k}$ with $Te_{n}=x _{n}$ where $e^{n}\,\,(n\in \mathbb{N})$ the sequences with $e^{n}_{n}=1$ and $e^{n}_{k}=0 $ for $k\neq n$.

In the literature, the Fibonacci numbers are the numbers in the following integer sequence:

$$ 1, 1, 2, 3, 5, 8, 13, 21, 34, 55, 89, 144, \ldots \,. $$

The sequence $(f_{n})$ of Fibonacci numbers is given by the linear recurrence relations

$$ f_{0}=f_{1}=1\quad \textrm{and}\quad f_{n} = f_{n-1} + f_{n-2},\quad n\geq 2. $$

This sequence has many interesting properties and applications in arts, sciences and architecture. For example, the ratio sequence of Fibonacci numbers converges to the golden ratio which is important in sciences and arts.

In [5], the Fibonacci matrix $\widehat{F}= (\widehat{f}_{nk})$ obtained using the Fibonacci numbers were defined as follows:

1.2 $$\begin{aligned}& \widehat{f}_{nk}= \textstyle\begin{cases} -\frac{f_{n+1}}{f_{n}}, & \textrm{if } k=n-1, \\ \frac{f_{n}}{f_{n+1}}, & \textrm{if } k=n, \\ 0, & \textrm{if } 0\leq k< n-1 \textrm{ or } k>n, \end{cases}\displaystyle \end{aligned}$$

for all $k,n\in \mathbb{N}$, and studied some topological properties of the sequence space $\ell_{p}(\widehat{F})$ for $1\leq p<\infty $. Later, in [6] the sequence spaces $\ell_{\infty }(\widehat{F})$ and $c_{0}(\widehat{F})$ were introduced as follows:

$$\begin{aligned} \ell_{\infty }(\widehat{F})= \biggl\{ x=(x_{n})\in w: \sup _{n}\biggl\vert \frac{f _{n}}{f_{n+1}}x_{n}- \frac{f_{n+1}}{f_{n}}x_{n-1}\biggr\vert < \infty \biggr\} \end{aligned}$$

and

$$\begin{aligned} c_{0}(\widehat{F})= \biggl\{ x=(x_{n})\in w: \lim _{n\rightarrow \infty } \biggl( \frac{f_{n}}{f_{n+1}}x_{n}- \frac{f_{n+1}}{f_{n}}x_{n-1} \biggr) =0 \biggr\} . \end{aligned}$$

Also in [7–14], many authors have defined and studied some new sequence spaces by using the matrix domain of a triangle infinite matrix.

In [15], for a sequence $x=(x_{k})$ in a normed space *X* the spaces $S(x)$ and $S_{w}(x)$ were defined by the set of all sequences $a=(a_{i})\in \ell_{\infty }$ such that $(a_{i}x_{i})\in \operatorname{cs}(X)$ and $(a_{i}x_{i})\in \operatorname{wcs}(X)$, respectively and several types of convergence of a series in a normed space have been characterized via these spaces. The completeness and barreledness of a normed space can also be characterized by means of the sequence spaces obtained by series in a normed space in [16] and [17, 18]. The characterizations of wucs are studied on locally convex spaces in [19].

In this paper, we introduce the sets $S\widehat{F}(x)$, $S\widehat{F} _{w}(x)$ and $S\widehat{F}_{w}^{*}(g)$ by means of sequences in normed spaces and the Fibonacci matrix $\widehat{F}= (\widehat{f}_{nk})$. We will characterize wucs by means of completeness of the spaces $S\widehat{F}(x)$ and $S\widehat{F}_{w}(x)$, and we will obtain necessary and sufficient conditions for the operator $T:S\widehat{F}(x) (\textrm{ and } S\widehat{F}_{w}(x))\rightarrow X$ to be continuous. Finally, we will give a characterization of the barreledness of a normed space through w^∗^ucs in $X^{*}$.

## Main results

Let $x= {x_{k}}$ and $g=(g_{k})$ be sequences in normed spaces *X* and $X^{*}$, respectively. We introduce the subspaces of $\ell_{\infty }( \widehat{F})$ which are defined by

$$\begin{aligned} S\widehat{F}(x) =& \biggl\{ a=(a_{k})\in \ell_{\infty }( \widehat{F}): \sum_{k}\widehat{F}(a_{k})x_{k}\textrm{ exists} \biggr\} , \end{aligned}$$

$$\begin{aligned} S\widehat{F}_{w}(x) =& \biggl\{ a=(a_{k})\in \ell_{\infty }(\widehat{F}): w-\sum_{k} \widehat{F}(a_{k})x_{k}\textrm{ exists} \biggr\} , \end{aligned}$$

and

$$\begin{aligned} S\widehat{F}_{w^{*}}(g) =& \biggl\{ a=(a_{k})\in \ell_{\infty }( \widehat{F}): w^{*}-\sum _{k}\widehat{F}(a_{k})g_{k}\textrm{ exists} \biggr\} , \end{aligned}$$

endowed with sup norm, where $w-\sum_{k}\widehat{F}(a_{k})x_{k}$ and $w^{*}-\sum_{k}\widehat{F}(a_{k})g_{k}$ define the limit in the weak topology and in the weak^∗^ topology, respectively.

In the following theorem we obtain a sufficient condition for the space $S\widehat{F}(x)$ to be a Banach space.

### Theorem 2.1

*Let* *X* *be a normed space and* $x=(x_{k})$ *be a sequence in X*. *If*

(i) *X* *is a Banach space*,
(ii) $x\in \operatorname{wuCs}(X)$,

*then* $S\widehat{F}(x)$ *is a Banach space*.

### Proof

Since $x\in \operatorname{wuCs}(X)$, the set *E* given in (1.1), is bounded. Therefore, there exists $M>0$ such that $\Vert E \Vert \leq M$. Let $(a^{m})$ be a Cauchy sequence in $S\widehat{F}(x)$. Since $\ell_{\infty }( \widehat{F})$ is a Banach space, there exists $a=(a_{k}^{0})\in \ell_{\infty }(\widehat{F})$ such that $\lim_{m} a^{m}=a^{0}$ in $\ell_{\infty }(\widehat{F})$. We will show that $a^{0}\in S \widehat{F}(x)$.

For $\epsilon >0$ there exists $m_{0}\in \mathbb{N}$ such that for every $m\geq m_{0}$ and $k\in \mathbb{N}$

$$\begin{aligned} \bigl\vert \widehat{F}\bigl(a^{m}_{k}\bigr)-\widehat{F} \bigl(a^{0}_{k}\bigr)\bigr\vert < \frac{ \epsilon }{3M}. \end{aligned}$$

Since $\frac{3M}{\epsilon }\vert \widehat{F}(a^{m})-\widehat{F}(a^{0}) \vert <1$, then $\frac{3M}{\epsilon }\sum_{k=1}^{n} ( \widehat{F}(a^{m}_{k})- \widehat{F}(a^{0}_{k}) ) x_{i}\in E$ and hence for $m>m_{0}$

$$\begin{aligned} \Biggl\Vert \sum_{k=1}^{n} \bigl( \widehat{F}\bigl(a^{m}_{k}\bigr)-\widehat{F} \bigl(a^{0} _{k}\bigr) \bigr) x_{k}\Biggr\Vert < \frac{\epsilon }{3}. \end{aligned}$$

Since $(a^{m})$ is a Cauchy sequence in $S\widehat{F}(x)$ there exists a sequence $(y_{m})\subset X$ such that for $n\geq n_{0}$

$$\begin{aligned} \Biggl\Vert \sum_{k=1}^{n}\widehat{F} \bigl(a^{m}_{k}\bigr)x_{k}-y_{m}\Biggr\Vert < \frac{ \epsilon }{3} \end{aligned}$$

and hence for $p>q>m_{0}$ and $n\in \mathbb{N}$ we have

$$\begin{aligned} \Vert y_{p}-y_{q} \Vert < \epsilon . \end{aligned}$$

Therefore $(y_{m})$ is a Cauchy sequence in *X*. Then for $\epsilon >0$ there exists $y_{0}\in X$ such that for $m>m_{1}$

$$\begin{aligned} \Vert y_{m}-y_{0} \Vert < \frac{\epsilon }{3}. \end{aligned}$$

If we suppose that $m_{2}=\max \{m_{0},m_{1}\}$, from the above inequalities, then we have

$$\begin{aligned} \Biggl\Vert \sum_{k=1}^{n}\widehat{F} \bigl(a^{0}_{k}\bigr)x_{k}-y_{0}\Biggr\Vert &\leq \Biggl\Vert \sum_{k=1}^{n} \bigl( \widehat{F}\bigl(a^{0}_{k}\bigr)-\widehat{F} \bigl(a^{m} _{k}\bigr) \bigr) x_{k}\Biggr\Vert \\ &\quad {}+\Biggl\Vert \sum_{k=1}^{n}\widehat{F} \bigl(a^{m}_{k}\bigr)x_{k}-y_{m}\Biggr\Vert +\Vert y _{m}-y_{0} \Vert \\ &< \frac{\epsilon }{3}+\frac{\epsilon }{3}+\frac{\epsilon }{3}=\epsilon . \end{aligned}$$

Consequently, $a^{0}\in S\widehat{F}(x)$. □

### Remark 2.2

Now, we will see that if the space $S\widehat{F}(x)$ is complete, then $c_{0}(\widehat{F})\subseteq S\widehat{F}(x)$. If we suppose that $c_{0}(\widehat{F})\nsubseteq S\widehat{F}(x)$, then there exists a sequence $a^{0}=(a_{k}^{0})\in c_{0}(\widehat{F})$ such that $\sum_{k}\widehat{F}(a^{0}_{k})x_{k}$ is not convergent. Since $c_{0}(\widehat{F})$ is a AD-space, there exists a Cauchy sequence $a=(a_{k}^{m})$ in $c_{00}$ (also in $S\widehat{F}(x)$) such that

$$ \lim_{m\rightarrow \infty }a_{k}^{m}=a_{k}^{0}. $$

Then $S\widehat{F}(x)$ is not complete.

The theorem that follows gives us a characterization of wucs.

### Theorem 2.3

*Let* *X* *is a normed space and* $x=(x_{k})$ *be a sequence in X*. *If* *X* *is a Banach space*, *then* $x\in \operatorname{wuCs}(X)$ *if and only if* $S \widehat{F}(x)$ *is a Banach space*.

### Proof

We prove that if $S\widehat{F}(x)$ is a Banach space, then $x\in \operatorname{wuCs}(X)$. Let us assume that $x\notin \operatorname{wuCs}(X)$. Then there exists a $g\in X^{*}$ such that $\sum_{k} \vert g(x_{k}) \vert =\infty $. We will construct a sequence that is $a=(a_{k})\in c_{0}({\widehat{F}}) \setminus S\widehat{F}(x)$. Let us choose $m_{1}\in \mathbb{N}$ such that $\sum_{k=1}^{m_{1}} \vert g(x_{k}) \vert >2.2$. We define

$$\begin{aligned}& a_{k}= \left \{ \textstyle\begin{array} {l@{\quad}l} \frac{1}{2} \sum_{i=0}^{k} \frac{f_{k+1}^{2}}{f_{i}f_{i+1}} , & \textrm{if } g(x_{k})\geq 0, \\ -\frac{1}{2} \sum_{i=0}^{k} \frac{f_{k+1}^{2}}{f_{i}f_{i+1}} , &\textrm{if } g(x_{k})< 0, \end{array}\displaystyle \right . \end{aligned}$$

for $k\in \{1,2, \ldots ,m_{1}\}$. Analogously, we can choose $m_{2}>m_{1}$ such that $\sum_{k=m_{1}+1}^{m_{2}} \vert g(x_{k}) \vert >3.3$, and we can also define

$$\begin{aligned}& a_{k}= \left \{ \textstyle\begin{array} {l@{\quad}l} \frac{1}{3} \sum_{i=0}^{k} \frac{f_{k+1}^{2}}{f_{i}f_{i+1}} , & \textrm{if } g(x_{k})\geq 0, \\ -\frac{1}{3} \sum_{i=0}^{k} \frac{f_{k+1}^{2}}{f_{i}f_{i+1}} , &\textrm{if } g(x_{k})< 0, \end{array}\displaystyle \right . \end{aligned}$$

for $k\in \{m_{1}+1, \ldots ,m_{2}\}$. Following this way, we obtain an increasing sequence $(m_{k})$ in $\mathbb{N}$ and the sequence $a=(a_{k})\in c_{0}(\widehat{F})$ such that

$$\begin{aligned} \sum_{k=1}^{\infty }\widehat{F}(a_{k})g(x_{k})= \infty . \end{aligned}$$

Then $a\notin S\widehat{F}(x)$ and hence $c_{0}(\widehat{F})\nsubseteq S\widehat{F}(x)$. From Remark 2.2, $S\widehat{F}(x)$ is not a Banach space. □

### Remark 2.4

If *X* is not Banach space, then the above theorem is not satisfied. Actually, If *X* is not Banach space then there exists a sequence $x=(x_{k})\in \ell_{1}(X)\setminus cs(X)$ such that for every $k\in \mathbb{N}$ and $x^{**}\in X^{**}\setminus X$

$$\begin{aligned} \Vert x_{k} \Vert < \frac{1}{k2^{k}} \quad \textrm{and}\quad\sum_{k} x_{k}=x ^{**}. \end{aligned}$$

We define the sequence $y=(y_{k})$ by

$$\begin{aligned}& y_{k}= \left \{ \textstyle\begin{array} {l@{\quad}l} kx_{k}, & \textrm{if } k \textrm{ odd}, \\ -kx_{k}, & \textrm{if } k \textrm{ even}. \end{array}\displaystyle \right . \end{aligned}$$

It obvious that $y=(y_{k})\in \operatorname{wuCs}(X)$. On the other hand, we consider the sequence $a=(a_{k})\in c_{0}({\widehat{F}})$ such that

$$\begin{aligned}& a_{k}= \left \{ \textstyle\begin{array} {l@{\quad}l} \frac{1}{2} \sum_{i=0}^{k} \frac{1}{k} \frac{f_{k+1}^{2}}{f_{i}f_{i+1}}, & \textrm{if } k \textrm{ odd}, \\ -\frac{1}{2} \sum_{i=0}^{k} \frac{1}{k} \frac{f_{k+1}^{2}}{f_{i}f_{i+1}}, & \textrm{if } k \textrm{ even}. \end{array}\displaystyle \right . \end{aligned}$$

Then $\sum_{k}\widehat{F}(a_{k})y_{k}=\frac{1}{2}x^{**}\in X^{**} \setminus X$. Therefore $a\notin S\widehat{F}(y)$ and hence $c_{0}({\widehat{F}})\nsubseteq S\widehat{F}(y)$. This shows that the space $S\widehat{F}(y)$ is not complete.

### Theorem 2.5

*Let* *X* *be a normed space and* $x=(x_{k})$ *be a sequence in X*. *We also define the linear operator*

$$\begin{aligned} T:S\widehat{F}(x) \rightarrow &X, \\ a \rightarrow & T(a)=\sum_{k} \widehat{F}(a_{k})x_{k}. \end{aligned}$$

*Then* *T* *is continuous if and only if* $x=(x_{k})\in \operatorname{wuCs}(X)$.

### Proof

If the operator *T* is continuous, then we prove that $x=(x_{k}) \in \operatorname{wuCs}(X)$. Since *T* is continuous, there exists $K>0$ such that $\Vert T(a_{k}) \Vert \leq K\Vert (a_{k}) \Vert $ for $a=(a_{k})\in S\widehat{F}(x)$.

Let $b=(b_{k})\in B_{c_{00}}$. Then there exists a sequence $a=(a_{k})\in c_{00}(\widehat{F})$ such that $\widehat{F}(a_{k})=b _{k}$ for every $k\in \mathbb{N}$. Since $c_{00}\subseteq S \widehat{F}(x)$, we have

$$\begin{aligned} \Biggl\Vert \sum_{k=1}^{n}b_{k}x_{k} \Biggr\Vert =\Biggl\Vert \sum_{k=1}^{n} \widehat{F}(a_{k})x_{k}\Biggr\Vert \leq K\bigl\Vert (a_{k}) \bigr\Vert . \end{aligned}$$

Therefore the set *E*, defined in (1.1), is bounded and hence $x=(x_{k})\in \operatorname{wuCs}(X)$.

Conversely, let $x\in \operatorname{wuCs}(X)$. Since the set *E* is bounded, there exists $K>0$ such that $\Vert e \Vert < K$ for every $e\in E$. If we take $(a_{k})\in S\widehat{F}(x)$, then

$$\begin{aligned} \Biggl\Vert \sum_{k=1}^{n} \frac{\widehat{F}(a_{k})}{\Vert \widehat{F}(a_{k}) \Vert }x_{k}\Biggr\Vert \leq K \end{aligned}$$

for $n\in \mathbb{N}$. Thus, *T* is continuous. □

Now, we will extend some of the above results to weak topology. First, let us start with the following result.

### Theorem 2.6

*Let* *X* *be a Banach space and* $x=(x_{k})$ *be a sequence in X*. *If* $x\in \operatorname{wuCs}(X)$, *then* $S\widehat{F}_{w}(x)$ *is a Banach space*.

### Proof

In the first place, as in Theorem 2.1, since $x\in \operatorname{wuCs}(X)$, we suppose that $\Vert e \Vert \leq M$ for every $e\in E$ and $(a^{m})$ be a Cauchy sequence in $S\widehat{F}_{w}(x)$ such that $a^{m}\rightarrow a^{0} \in \ell_{\infty }(\widehat{F})$, as $m\rightarrow \infty $.

Let $\epsilon >0$ and let $m_{0}\in \mathbb{N}$ such that for every $m\geq m_{0}$ and $k\in \mathbb{N}$

$$\begin{aligned} \bigl\vert \widehat{F}\bigl(a^{m}_{k}\bigr)-\widehat{F} \bigl(a^{0}_{k}\bigr)\bigr\vert < \frac{ \epsilon }{3M}. \end{aligned}$$

Since $\frac{3M}{\epsilon }\vert \widehat{F}(a^{m})-\widehat{F}(a^{0}) \vert <1$, $\frac{3M}{\epsilon }\sum_{k=1}^{n} ( \widehat{F}(a^{m}_{k})- \widehat{F}(a^{0}_{k}) ) x_{i}\in E$ and hence for $m>m_{0}$

$$\begin{aligned} \Biggl\Vert \sum_{k=1}^{n} \bigl( \widehat{F}\bigl(a^{m}_{k}\bigr)-\widehat{F} \bigl(a^{0} _{k}\bigr) \bigr) x_{k}\Biggr\Vert < \frac{\epsilon }{3}. \end{aligned}$$

Since $(a^{m})$ is a Cauchy sequence in $S\widehat{F}_{w}(x)$ there exists a sequence $(y_{m})\subset X$ such that for $n\geq n_{0}$ and for all $f\in X^{*}$

$$\begin{aligned} \Biggl\vert \sum_{k=1}^{n}\widehat{F} \bigl(a^{m}_{k}\bigr)f(x_{k})-f(y_{m}) \Biggr\vert < \frac{ \epsilon }{3}. \end{aligned}$$

From the Hahn-Banach theorem there exists a functional *f* in $X^{*}$ such that

$$ \Vert y_{p}-y_{q} \Vert =\bigl\vert f(y_{p}-y_{q}) \bigr\vert . $$

Then we have

$$ \Vert y_{p}-y_{q} \Vert < \epsilon $$

for $p>q>m_{0}$ and $n\in \mathbb{N}$, and hence $(y_{m})$ is a Cauchy sequence in *X*. Let us suppose that $y_{0}\in X$ such that for $m>m_{1}$

$$\begin{aligned} \Vert y_{m}-y_{0} \Vert < \frac{\epsilon }{3}. \end{aligned}$$

If we take $m_{2}=\max \{m_{0},m_{1}\}$, then we have

$$\begin{aligned} \Biggl\vert \sum_{k=1}^{n}\widehat{F} \bigl(a^{0}_{k}\bigr)f(x_{k})-f(y_{0}) \Biggr\vert & \leq \Biggl\vert \sum_{k=1}^{n} \bigl( \widehat{F}\bigl(a^{0}_{k}\bigr)-\widehat{F}\bigl(a ^{m}_{k}\bigr) \bigr) f(x_{k})\Biggr\vert \\ &\quad {}+\Biggl\vert \sum_{k=1}^{n}\widehat{F} \bigl(a^{m}_{k}\bigr)f(x_{k})-f(y_{m}) \Biggr\vert +\bigl\vert f(y_{m})-f(y_{0}) \bigr\vert \\ &< \frac{\epsilon }{3}+\frac{\epsilon }{3}+\frac{\epsilon }{3}=\epsilon . \end{aligned}$$

Therefore, $a^{0}\in S\widehat{F}_{w}(x)$. □

### Theorem 2.7

*Let* *X* *be a normed space and* $x=(x_{k})$ *be a sequence in X*. *If* *X* *is a Banach space*, *then* $x\in \operatorname{wuCs}(X)$ *if and only if* $S \widehat{F}_{w}(x)$ *is a Banach space*.

### Proof

By Theorem 2.6, it is enough to show that if $S\widehat{F}_{w}(x)$ is a Banach space, then $x\in \operatorname{wuCs}(X)$. We suppose that there exists a $g\in X^{*}$ such that $\sum_{k} \vert g(x_{k}) \vert = \infty $. Similarly as in the proof of Theorem 2.3, we can construct a sequence $a=(a_{k})\in c_{0}(\widehat{F})$ such that

$$\begin{aligned} \sum_{k=1}^{\infty }\widehat{F}(a_{k})g(x_{k})= \infty . \end{aligned}$$

From the definition of $S\widehat{F}_{w}(x)$, we have $a=(a_{k}) \notin S\widehat{F}_{w}(x)$. Then $S\widehat{F}_{w}(x)$ is not complete. □

### Theorem 2.8

*Let* *X* *be a normed space and* $x=(x_{k})$ *be a sequence in X*. *We also define the linear operator*

$$\begin{aligned} T:S\widehat{F}_{w}(x) \rightarrow &X, \\ a \rightarrow & T(a)=w-\sum_{k} \widehat{F}(a_{k})x_{k}. \end{aligned}$$

*Then* *T* *is continuous if and only if* $x=(x_{k})\in \operatorname{wuCs}(X)$.

### Proof

The proof is similar to that of Theorem 2.5. □

For a normed space *X* and a sequence $g=(g_{i})$ in $X^{*}$, the set $S\widehat{F}_{w^{*}}(g)$ was defined by

$$\begin{aligned} S\widehat{F}_{w^{*}}(g) =& \biggl\{ a=(a_{k})\in \ell_{\infty }( \widehat{F}): w^{*}-\sum _{k}\widehat{F}(a_{k})g_{k}\textrm{ exists} \biggr\} . \end{aligned}$$

The next theorem shows that if the normed space *X* is barreled, then weakly unconditionally Cauchy series and weakly^∗^ unconditionally Cauchy series in $X^{*}$ are equivalent.

### Theorem 2.9

*Let* *X* *be a normed space and* $g=(g_{i})$ *be a sequence in* $X^{*}$. *Consider the following statements*:

(i) $g\in \operatorname{wuCs}(X^{*})$.
(ii) $S\widehat{F}_{w^{*}}(g)=\ell_{\infty }(\widehat{F})$.
(iii) $g\in w^{*}\operatorname{ucs}(X^{*})$; *that is*, $\sum_{k}\vert g_{k}(x) \vert < \infty $ *for every* $x\in X$.

*We have* (i) ⇒ (ii) ⇒ (iii). *Furthermore* *X* *is a barreled normed space if and only if the three conditions are equivalent*.

### Proof

(i) ⇒ (ii). Since $S\widehat{F}_{w^{*}}(g)\subset \ell_{\infty }(\widehat{F})$, we will show that $\ell_{\infty }( \widehat{F})\subset S\widehat{F}_{w^{*}}(g)$. If $a=(a_{k})\in \ell_{\infty }(\widehat{F})$, then $(\widehat{F}(a_{k})g_{k})\in \operatorname{wuCs}(X ^{*})$. Thus $\sum_{k=1}^{\infty }\widehat{F}(a_{k})g_{k}$ is weak^∗^ convergent in $X^{*}$ and hence $a=(a_{k})\in S\widehat{F}_{w ^{*}}(g)$.

(ii) ⇒ (iii). It is obvious.

If *X* is a barreled space then we will show that (iii) ⇒ (i). We define the set $E'$ by

$$\begin{aligned} E'= \Biggl\{ \sum_{k=1}^{n}a_{k}g_{k}: \, \vert a_{k} \vert \leq 1,\,k=1,2,\ldots ,n; \, n\in \mathbb{N} \Biggr\} . \end{aligned}$$

It is easily see that the set $E'$ is pointwise bounded. Since *X* is barreled, $E'$ is bounded for the norm topology of $X^{*}$. Therefore $(g_{k})\in \operatorname{wuCs}(X^{*})$.

Conversely, if (iii) ⇒ (i) are equivalent, then we will prove that *X* is a barreled space. Let us suppose that *X* is not a barreled space. Then there exists a weak^∗^-bounded set $N\subseteq X^{*}$ which is not bounded. Let $(g_{k})\in N$ such that $\Vert g_{k} \Vert >2^{k}.2^{k}$ for $k\in \mathbb{N}$. If we take $h_{k}= \frac{1}{2^{k}}g_{k}$ for $k\in \mathbb{N}$ then it is clear that $(h_{k}(x))\in \ell_{1}$ for every $x\in X$. Since $\Vert h_{k} \Vert >2^{k}$ for every $k\in \mathbb{N}$, the series $\sum_{k=1}^{\infty } \frac{1}{2^{k}}h_{k}$ does not convergence. Hence $(h_{k})\notin \operatorname{wuCs}(X ^{*})$. □

## Conclusion

In this paper, we introduced and studied the sets $S\widehat{F}(x)$, $S\widehat{F}_{w}(x)$ and $S\widehat{F}_{w}^{*}(g)$ via sequences in normed spaces and the Fibonacci matrix $\widehat{F}= (\widehat{f}_{nk})$. We obtained the characterizations of continuous linear operator and weakly unconditionally Cauchy series by means of completeness of the space $S\widehat{F}(x)$, and we extended the obtained results to weak topology. Also, we gave necessary and sufficient conditions for a normed space *X* to be barreled space. Furthermore, one can obtain more general conclusion corresponding to the results of this paper by taking more general matrices instead of the Fibonacci matrix.

## References

[1] Albiac F, Kalton NJ (2006). Topics in Banach Spaces Theory.

[2] Diestel J (1984). Sequences and Series in Banach Spaces.

[3] Bessaga C, Pelczynski A (1958). On bases and unconditional convergence of series in Banach spaces. Stud. Math..

[4] McArthur CW (1956). On relationships amongst certain spaces of sequences in an arbitrary Banach space. Can. J. Math..

[5] Kara EE (2013). Some topological and geometrical properties of new Banach sequence spaces. J. Inequal. Appl..

[6] Başarır, M, Başar, F, Kara, EE: On the spaces of Fibonacci difference null and convergent sequences. arXiv:1309.0150v1 [math.FA] (2013)

[7] Alotaibi A, Mursaleen M, Alamri BAS, Mohiuddine SA (2015). Compact operators on some Fibonacci difference sequence spaces. J. Inequal. Appl..

[8] Altay B, Başar F (2007). Certain topological properties and duals of the domain of a triangle matrix in a sequence space. J. Math. Anal. Appl..

[9] Başar F, Altay B (2003). On the space of sequences of p-bounded variation and related matrix mappings. Ukr. Mat. Zh..

[10] Kara EE, Başarır M, Mursaleen M (2015). Compactness of matrix operators on some sequence spaces derived by Fibonacci numbers. Kragujev. J. Math..

[11] Karakaya V, Savaş E, Polat H (2013). Some paranormed Euler sequence spaces of difference sequences of order *m*. Math. Slovaca.

[12] Kirişçi M, Başar F (2010). Some new sequence spaces derived by the domain of generalized difference matrix. Comput. Math. Appl..

[13] Malkowsky E, Rakočević V (2007). On matrix domains of triangles. Appl. Math. Comput..

[14] Mursaleen M, Noman AK (2010). On the spaces of *λ*-convergent and bounded sequences. Thai J. Math..

[15] Aizpuru A, Pérez-Fernández FJ (1999). Characterizations of series in Banach spaces. Acta Math. Univ. Comen..

[16] Pérez-Fernández FJ, Benítez-Trujillo F, Aizpuru A (2000). Characterizations of completeness of normed spaces through weakly unconditionally Cauchy series. Czechoslov. Math. J..

[17] Kama, R, Altay, B: On some new characterizations of completeness and barrelledness of normed spaces. Contemp. Anal. Appl. Math. (2017, in press)

[18] Kama, R, Altay, B: Some sequence spaces and completeness of normed spaces. Creat. Math. Inform. (2017, in press)

[19] Ronglu L, Cho M-H (1995). Weakly unconditional Cauchy series on locally convex spaces. Northeast. Math. J..

